# Examining the Relationship between Free Sugars and Calorie Contents in Canadian Prepacked Foods and Beverages

**DOI:** 10.3390/foods6090075

**Published:** 2017-09-05

**Authors:** Jodi T. Bernstein, Wendy Lou, Mary R. L’Abbe

**Affiliations:** 1 Department of Nutritional Sciences, Faculty of Medicine, University of Toronto, FitzGerald Building, 150 College Street, Toronto, ON M5S 3E2, Canada; jodi.bernstein@mail.utoronto.ca; 2 Biostatistics Division, Dalla Lana School of Public Health, University of Toronto, Health Sciences Building, 155 College Street, Toronto, ON M5T 3M7, Canada; wendy.lou@utoronto.ca

**Keywords:** sugars, free sugars, calories, food supply, nutrient composition, food reformulation

## Abstract

To align with broader public health initiatives, reformulation of products to be lower in sugars requires interventions that also aim to reduce calorie contents. Currently available foods and beverages with a range of nutrient levels can be used to project successful reformulation opportunities. The objective of this study was to examine the relationship between free sugars and calorie levels in Canadian prepackaged foods and beverages. This study was a cross-sectional analysis of the University of Toronto’s 2013 Food Label Database, limited to major sources of total sugar intake in Canada (*n* = 6755). Penalized B-spline regression modelling was used to examine the relationship between free sugar levels (g/100 g or 100 mL) and caloric density (kcal/100 g or 10mL), by subcategory. Significant relationships were observed for only 3 of 5 beverage subcategories and for 14 of 32 food subcategories. Most subcategories demonstrated a positive trend with varying magnitude, however, results were not consistent across related subcategories (e.g., dairy-based products). Findings highlight potential areas of concern for reformulation, and the need for innovative solutions to ensure free sugars are reduced in products within the context of improving overall nutritional quality of the diet.

## 1. Introduction

Free sugars, the sugars that have been removed from their naturally-occurring sources (i.e., removed from whole fruits, vegetables, dairy products, and some grains) [1] are often found in foods that are both energy-dense and nutrient-poor [2]. Isolated from their naturally-occurring sources, free sugars become readily available to add into foods and beverages (“added sugars”), and to consume in greater quantities compared to the still intact intrinsic sugars, which are eaten as part of a healthy, balanced diet [3]. The association between excess free sugars consumption and increased risk of obesity, diabetes, and cardiovascular disease is thought to be mediated, at least in part, through energy intake and the metabolism of sugars [4,5,6,7]. In response, guidance from the WHO and other organizations have emerged, recommending intakes of free sugars be limited to a maximum 10% of calories [1,8,9,10]. As a proportion of energy intake, this recommendation can be tailored to any level of energy consumption, be presented as an absolute amount for use on nutrition labels [11,12], or be used to evaluate the proportion of calories within a food or beverage coming from free sugars [13]. Given the past successes of product reformulation for trans fat and sodium in Canada [14], the UK [15], and several other countries, a similar program to lower free sugar contents, is a viable option to consider to meet free sugars intake recommendations [16].

A previous study found that an average of 20% of calories in Canadian prepackaged foods and beverages came from free sugars [17], far in excess of the recommended maximum proportion [13]. Additionally, products within a food category had a wide range of free sugar levels, indicating that foods and beverages with lower free sugar levels are achievable, acceptable, and available [17]. However, reformulating products to be lower in free sugars requires interventions that also aim to reduce overall calorie contents whenever possible [18]. Removing free sugars, especially in solid foods, often requires replacement with other ingredients to maintain bulk, texture, and other functional properties of sugars [19]. This additional complexity means reductions in free sugar levels may not always result in a reduction in calories [20].

It is essential to determine if products that are lower in free sugars have more favourable calorie levels to inform any future reformulation strategies. Limited research has been conducted in this area and therefore this study aimed to examine the relationship between free sugars and calorie levels in major sources of foods and beverages contributing to total sugars intakes in Canada.

## 2. Materials and Methods

### 2.1. Food Composition Database

This study was a cross-sectional analysis of the University of Toronto’s Food Label Information Program (FLIP) 2013 database (*n* = 15,342). FLIP 2013 contains information (nutrient contents, Ingredient List, Universal Product Code, price, etc.) on Canadian prepackaged foods and beverages collected in 2013 for private-label and national brand foods. The database is described in detail elsewhere [17]. Excluded from this analysis was meal replacement beverages, which are indicated for special dietary use (*n* = 55), products with missing total sugar declarations (*n* = 28), and items with errors in nutrient declarations (*n* = 55), determined with Atwater calculations that varied by >20% from declared caloric values.

This analysis was further limited to the types of prepackaged foods and beverages that were top sources of total sugar intake (contributed to 91% of consumption), based on the national Canadian Community Health Survey 2.2, Nutrition Cycle (CCHS 2004) data [21]. Top sources were divided into sugar-focused subcategories according to a previously developed taxonomy [17]. The taxonomy was based on Schedule M food categories as outlined in the Canadian Food and Drug Regulations [22], as well as Health Canada’s sodium-focused categories [23]. Subcategories excluded from this analysis include sugar (table sugar) (*n* = 7), frozen fruits (*n* = 61), other beverages (e.g., tea from tea bags, coffee from grounds) (*n* = 33), vegetable pastes (*n* = 20), and flavoured water (*n* = 55), as these subcategories had no variation in free sugar content, with the former containing 100% free sugars and the latter subcategories containing 0%. Remaining products (*n* = 6755) were evaluated as part of 37 subcategories; 32 food subcategories and 5 beverage subcategories (see Table 1 for subcategory descriptions).

### 2.2. Free Sugars and Calorie Levels

Free sugar contents (g/100 g or 100 mL) were calculated using the University of Toronto’s free sugar algorithm [17]. Calorie contents for each product were obtained from the Nutrition Facts table as per the manufacturers stated serving size and converted to standardized units (kcal/100 g or 100 mL). ESHA Food Processor software and food composition data from the Canadian Nutrient File [24] were used to calculate nutrient values for products requiring preparation, to facilitate comparisons with ready-to-consume items. Free sugars and calorie contents are presented per 100 mL for beverages and select dessert subcategories (see Table 1) and per 100 g for the remaining subcategories (see Table 1).

## 3. Statistical Analysis

To our knowledge there has been no previous examination of the relationship between free sugars and calories in prepackaged foods and beverages that could be used to guide this analysis. One of our main concerns was possible nonlinearity. To allow for this possibility, several types of regression analyses were conducted including nonparametric procedures.

### 3.1. Linear & Polynomial Regression

Firstly, an exploration of the relationship between calories and free sugar levels for each subcategory was conducted through visual inspection of scatter plots to indicate potential applicability of parametric regression procedures. Based on examination of the scatter plots, linear and polynomial regressions were conducted for subcategories where it was deemed appropriate. Analyses were conducted using PROC REG (SAS Institute Inc., Cary, NC, USA) specifying the independent variable (i.e., free sugars content) and the dependent variable (i.e., calorie content). For polynomial regression, free sugars was squared to create a new variable which was added into the model. The relative fit of the linear and polynomial models was assessed based on the *p*-values and through inspection of the fit diagnostics.

### 3.2. Penalized Spline Regression

For all subcategories, splines, a semiparametric approach to modelling, was used to capture systematic deviations from the overall trend in the data [25]. Splines are comprised of segments of parametric functions that connect to create a continuous join [25]. For this study, penalized B-spline regression analyses were used. In general, B-splines are constructed from polynomial segments and they do not rely on assumptions about trends in the data [25,26]. Another consideration was that data-driven modeling has the potential to result in an overfitted model, optimized for the database it was drawn from, but unlikely to be generalizable. For this reason, penalized smoothing was used which automatically computes the optimal degree of smoothness to create robust regression models that are not overfitted [27]. Analysis was carried out using the GLIMMIX procedure specifying the independent variable (i.e., free sugars content) be modelled using penalized B-spline random effects transformations. All analyses were conducted in SAS 9.4 (SAS Institute Inc., Cary, NC, USA). A probability level of *p* < 0.05 was used to indicate statistical significance.

## 4. Results

### 4.1. Linear & Polynomial Regression

A visual inspection of the fit diagnostics for linear and polynomial regressions that had significant *p*-values (*p* < 0.05) showed that neither of these models provided a robust description of the changes in calories as a function of free sugar for any food or beverage subcategory.

### 4.2. Penalized Spline Regression

**Figure 1:**
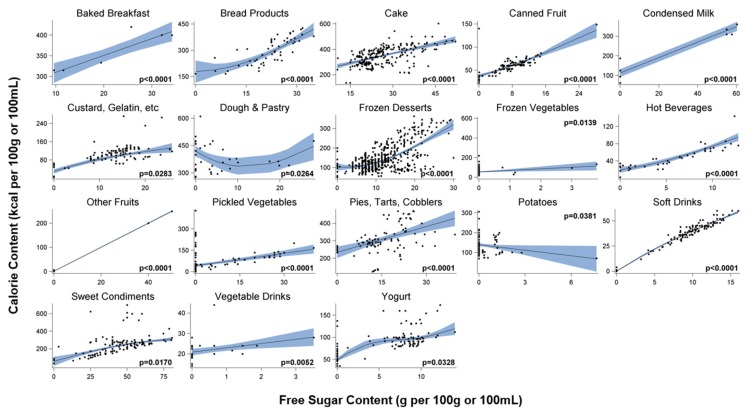
Semi-parametric estimates and observed calorie content (kcal per 100 g/100 mL) as a function of free sugar contents (g per 100 g/100 mL) for each food and beverage subcategory with significant results, as estimated with penalized B-splines. Dots show observed calorie contents. Solid lines show predicted mean curve. Blue bands show 95% confidence interval limits.

Figure 1 summarizes the penalized B-spline regression analyses for the food and beverage subcategories with significant results. Three of the five beverage subcategories and 14 of the 32 food subcategories, had a significant relationship between free sugars and calorie levels (Figure 1). Most significant models showed positive relationships between free sugars and calorie levels. The majority had increasing relationships without an apparent plateau and followed a near linear or slightly curvilinear pattern. There were only a few subcategories that deviated from this pattern. Analyses of *yogurts* showed periods in which the slope of the predicted mean curve fluctuated, although a positive relationship was maintained overall. Only two subcategories (e.g., *dough and pastry* and *potatoes*) exhibited a period of decline in calorie contents with increasing free sugar levels. The final incline in *dough and pastry* and the decline in *potatoes* appear to be driven largely by a few observations and there was a wider confidence interval around the predicted mean for these two subcategories compared to other significant food categories.

**Figure 2:**
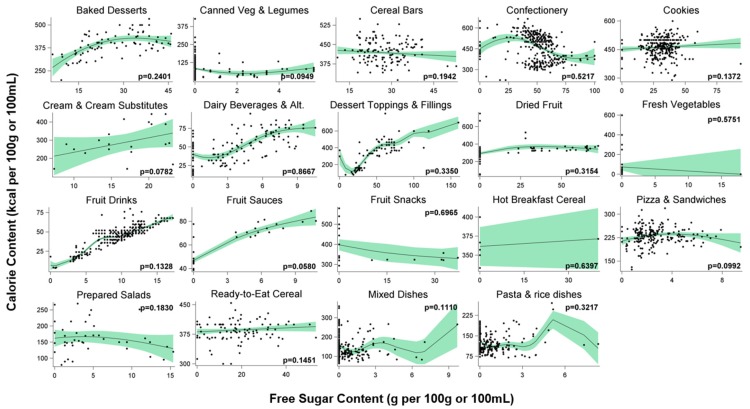
Semi-parametric estimates and observed calorie content (kcal per 100 g/100 mL) as a function of free sugar contents (g per 100 g/100 mL) for each food and beverage subcategory with non-significant results, as estimated with penalized B-splines. Dots show observed calorie contents. Solid lines show predicted mean curve. Green bands show 95% confidence interval limits.

Figure 2 summarizes the results for the remaining, non-significant, subcategories. Overall, more of the relationships in these subcategories followed irregular or flat trends.

Significance varied across the broader, related, food and beverage groups of sugar-sweetened beverages (i.e., *fruit drinks*, *hot beverages*, and *soft drinks),* dairy-based (i.e., *frozen desserts*, *condensed milk*, *dairy beverages and alternatives*, *yogurt*, and *cream and cream substitutes*), grain-based (i.e., *baked breakfast*, *baked desserts*, *bread products*, *cake*, *cereal bars*, *cookies*, *dough and pastry*, *pies*, *tarts*, *and cobblers*, *pasta and rice dishes*, and *pizza and sandwiches*), fruit-based (i.e., *canned fruit*, *dried fruit*, *fruit sauces*, *fruit snacks*, and *other fruits*), and vegetable-based products (i.e., *canned vegetables and legumes*, *fresh vegetables*, *frozen vegetables*, *pickled vegetables*, *potatoes*, *prepared salads*, and *vegetable drinks)*.

## 5. Discussion

Dietary guidelines recommending limiting intakes of free sugars as well as excess energy, need to be supported with the availability of suitable options in the food supply. This study was conducted to inform free sugars reformulation strategies. It is the first, to our knowledge, to examine the relationship between free sugar levels and caloric density among comparable prepackaged foods and beverages. Free sugars are added to foods to provide sweetness, but also for a variety of other functional reasons [19]. Replacing it is complicated by the need to find ingredients to satisfy all of sugars’ functional roles [19]. This analysis identified both food and beverage subcategories in which lower free sugars options are likely to have lower calorie contents and conversely, where calorie contents were not related to free sugar levels.

The ingredients used to replace free sugars may at least partially explain why some relationships were significant and others were not. For example, low or no-calorie sweeteners (LCS) can be used to create sugar-free alternatives. LCS, are often added as a direct replacement for sugars in sugar-sweetened beverages (e.g., *soft drinks*), resulting in less energy-dense alternatives. Beverages in this study with lower free sugars and calorie levels were primarily composed of water and “added” sugars, making them ideal candidates for LCS use. However, it was evident that such a simple substitution was not suitable for all beverages, which may explain why both significant and non-significant relationships were found. For instance, *fruit juices*, in which the free sugars are naturally-occurring, are difficult to reformulate because sugar that has not been “added” cannot be removed without compromising the products’ integrity as 100% fruit juice [28]. While LCS provide sweetness, it does not always the bulk required to replace free sugars in solid foods [28].

Although prior research has not specifically focused on the relationship between free sugars and calorie contents, there has been extensive research into the relationship between dietary sugars and fat intakes, dubbed the “sugar-fat see-saw” [29]. This theory has been explored substantially, and refers to increasing intakes or levels of fat corresponding to decreasing intakes or levels of sugars and vice versa [29]. Presumably, calorie contents would increase with declining free sugar contents if sugars are replaced with fats, which contribute more than double the calories per gram. Based on this presumption, there did not appear to be evidence of a sugar-fat see-saw in the prepackaged foods and beverages examined. For example, additional exploratory assessment of nutrition information and ingredient lists indicated that some of the calories from free sugars in lower free sugars products may have been exchanged with fats and/or proteins only in *dairy beverages and alternatives*, *baked desserts*, and *cream and cream substitutes*, or with carbohydrates in *pizzas and sandwiches* (data not shown). However, this study did not investigate this research question specifically.

Additionally, some of the variation in the observed relationships between free sugars and calorie levels may reflect variability in the types of products analyzed within a subcategory. For example, some minor categories in *baked desserts*, *cake*, *dairy beverages and alternatives*, *canned fruit*, *confectionery*, *frozen dessert*, and *condensed milk* clustered together and may have driven the relationships, at least in part. Differences in the relationships between free sugars and calorie levels may be more apparent when looking among specific minor categories.

Limitations of this study include those related to the calculation of free sugars and the use of nutrient information declared on the NFt, as discussed elsewhere [17]. The calculation of free sugar contents was analyzed for prepackaged foods with nutrition labels, based on an algorithm developed by Louie and colleagues, that has been shown to have high levels of inter-researcher repeatability [30]. Although this study examined sources of sugars in the diet, it was not weighted to be reflective of intakes, nor did this analysis include whole or unpackaged foods that contribute to total sugar intakes in Canada. The data used in this study was collected in 2013 and any changes to the food supply occurring since then are not reflected in the results. This study also did not quantify changes in ingredients responsible for the observed trends, although we did examine ingredient lists to help understand the relationships between calories and free sugar levels. Further research is needed to systematically assess the types of macronutrients and ingredients that contribute to the corresponding difference or lack of difference in calorie contents with varying free sugar levels, as well as to assess the significance of these relationships when comparing very specific types of products at the minor category level.

Strengths of this study include the evaluation of products that are major contributors to total sugar consumption. Thus, the analyzed food data is focused on where interventions and guidelines could be targeted and where there is greater potential for impacting free sugar intakes. In addition, this study addressed concerns that focusing on a single nutrient of public health concern may lead to unintended consequences, as seen with the demonization of all types of fats in the 1980s and 1990s [31]. By evaluating free sugars in the context of caloric density, this is accounted for.

In summary, these data provide the first assessment of the relationship between calorie contents and free sugar levels in the prepackaged foods and beverages that are top contributors to total sugar consumption in Canada. The results of this study are mixed, showing both significant and non-significant relationships. Ultimately, these findings indicate that there may be limited availability of lower free sugar alternatives that have relatively lower calorie contents in many food and beverage subcategories. This data can also be used to monitor possible areas of concern as the results of this study can shed light on what may be expected to happen should products be reformulated to be lower in free sugars. Limiting free sugar levels in foods and beverages is an important strategy to consider to reduce the detrimental effects associated with excess free sugar intakes. However, it is imperative that it be done within the context of improving the overall nutritional quality of the diet.

## Figures and Tables

**Table 1:** Examples of foods and beverages included in each subcategory evaluated (*n* = 6755).

Subcategory	*n*	Food and Beverage Examples
Baked Breakfast	11	Toaster pastries.
Baked Desserts	88	Brownies, squares, doughnuts, pastries, sweet buns.
Bread Products	57	Muffins, quick breads.
Cake	246	Cakes (e.g., cheesecake, cupcakes, snack cakes, coffee cake, fruit cake).
Canned Fruit	155	Fruit canned in juice; fruit canned in syrup; fruit canned in water.
Canned Vegetables & Legumes	457	Canned vegetables (e.g., tomatoes, corn, peas), canned legumes (e.g., beans).
Cereal Bars	202	Cereal and granola bars with and without fillings/toppings.
Condensed Milk	18	Condensed milk, evaporated milk.
Confectionery	466	Candies (e.g., mints, sprinkles, gummies, marshmallows), chocolate.
Cookies	411	Cookies (e.g., sandwich, chocolate chip, shortbread, wafer).
Cream & Cream Substitutes	21	Cream, whipped dessert toppings.
Custard, Gelatin, Pudding, etc. ^b^	195	Custard, pudding, mousse, gelatin.
Dairy Beverages & Alternatives ^a,b^	226	Plain and flavoured milk, plant-based milk, drinkable yogurt.
Dessert Toppings & Fillings ^b^	119	Dessert toppings and spreads (e.g., chocolate sauce), cake frosting and icing.
Dough & Pastry	49	Pie shells, crust.
Dried Fruit	150	Dried fruit, sweetened and unsweetened.
Fresh Vegetables	53	Fresh vegetables (e.g., pre-cut vegetables, pre-washed lettuce).
Frozen Desserts ^b^	624	Ice cream, ice milk, frozen yogurt, cones, bars, sandwiches, sundaes, sorbet, popsicles.
Frozen Vegetables	155	Frozen vegetables with and without sauce.
Fruit Drinks ^a,b^	648	Fruit drinks, fruit juice-drink combination beverages, 100% fruit juice.
Fruit Sauces	62	Sweetened and unsweetened fruit sauce (e.g., apple sauce).
Fruit Snacks	40	Apple chips, banana chips, fruit leather/bars, fruit-based gummies.
Hot Beverages ^a,b^	58	Sweetened coffee, cocoa, hot chocolate, tea.
Hot Breakfast Cereal	7	Cream of wheat.
Mixed Dishes	289	Mixed refrigerated and frozen dishes.
Other Fruits	12	Fruit garnishes (e.g., maraschino cherries), fruit juice as an ingredient (e.g., lemon juice).
Pasta & Rice Dishes	293	Pasta and rice dishes (shelf-stable, frozen meals, refrigerated meals, pasta salads).
Pickled Vegetables	180	Sweet and sour pickled vegetables.
Pies, Tarts, Cobblers, Crisps	98	Pies, tarts, crisps, pie filling (except cherry).
Pizza & Sandwiches	213	Frozen and refrigerated pizzas, hot dogs, sandwiches, burgers. Excludes individual components (e.g., buns, patties).
Potatoes	126	Mashed potatoes, scalloped potatoes, sweet potatoes, fries.
Prepared Salads	44	Vegetable salad, potato salad, pasta salad, coleslaw.
Ready-to-Eat Breakfast Cereal	137	Flaked, puffed, semi-compact cereal. Excludes high-fibre and shredded cereal.
Soft Drinks ^a,b^	271	Soft drinks, iced tea (diet, calorie-reduced, regular). Includes beverages requiring preparation.
Sweet Condiments	298	Honey, molasses, bread spreads (e.g., chocolate hazelnut spread), syrups, fruit preserves (e.g., jam, jelly).
Vegetable Drinks ^a,b^	43	Vegetable juice and cocktails (e.g., tomato-clam beverage).
Yogurt	233	Plain and flavoured yogurt.

^a^ Denotes beverage categories; ^b^ sugar and calorie contents are presented per 100 mL for beverages and select dessert subcategories. The remaining subcategories are presented per 100 g.
